# Production of diamond using intense heavy ion beams at the FAIR facility and application to planetary physics

**DOI:** 10.1038/s41598-023-28709-7

**Published:** 2023-01-26

**Authors:** Naeem Ahmad Tahir, Vincent Bagnoud, Paul Neumayer, Antonio Roberto Piriz, Sofia Ayelen Piriz

**Affiliations:** 1 GSI Helmholtzzentrum für Schwerionenforschung, Planckstraße 1, 64291 Darmstadt, Germany; 2 E.T.S.I. Industriales, Universidad de Castilla-La Mancha, 13071 Ciudad Real, Spain

**Keywords:** Planetary science, Astronomy and planetary science, Physics

## Abstract

Diamonds are supposedly abundantly present in different objects in the Universe including meteorites, carbon-rich stars as well as carbon-rich extrasolar planets. Moreover, the prediction that in deep layers of Uranus and Neptune, methane may undergo a process of phase separation into diamond and hydrogen, has been experimentally verified. In particular, high power lasers have been used to study this problem. It is therefore important from the point of view of astrophysics and planetary physics, to further study the production processes of diamond in the laboratory. In the present paper, we present numerical simulations of implosion of a solid carbon sample using an intense uranium beam that is to be delivered by the heavy ion synchrotron, SIS100, that is under construction at the Facility for Antiprotons and Ion Research (FAIR), at Darmstadt. These calculations show that using our proposed experimental scheme, one can generate the extreme pressure and temperature conditions, necessary to produce diamonds of mm^3^ dimensions.

## Introduction

Diamonds are ubiquitous in the Universe. Tiny diamonds (nanodiamonds) that contain up to 2000 carbon atoms, are abundant in meteorites, while some are formed in stars even before the Solar System existed^1^. It is also proposed that diamonds exist in carbon-rich stars, particularly in white dwarfs^2^. Moreover, it is expected that some carbon-rich, extrasolar planets may be almost pure diamond^3,4^. Since accessing such objects directly is obviously extremely challenging, complementary studies of the processes that lead to diamond production in the laboratory will be very helpful in understanding the formation and evolution of different heavenly objects. This can be achieved by subjecting carbon and its compounds to the extreme physical conditions that exits in the planetary interiors and stars that transform carbon into diamond. Another research area that will benefit from these studies is the investigation of the formation conditions of various stacking disordered carbon structures produced as a result of large asteroidal impacts. For instance, diamond structures called diaphites^5–7^, that have been discovered at the impact sites, Canyon Diablo and Popigai. Due to the special electronic and mechanical properties of these diamond forms, they may have high potential industrial significance, that underlines their importance.

High pressure experiments suggest that large amount of diamonds are formed from methane on the ice giant planets, Uranus and Neptune. In a recent experiment^8^, in which a polystyrene sample was dynamically compressed using a laser, extreme physical conditions that are expected to exist around 10,000 km below the surfaces of Uranus and Neptune, were achieved. These include a pressure of 150 GPa and a temperature of 5000 K. This experiment has demonstrated the carbon-hydrogen separation and diamond precipitation under these conditions. In another experiment^9^ that used a 100 femtosecond laser pulse to irradiated a sample of highly oriented pyrolytic graphite, formation of nanoscale cubic diamond crystals in laser-irradiated areas was observed.

Intense particle beams are now considered to be a new tool that can be used to generate extended samples of High Energy Density (HED) matter with fairly uniform conditions. It is interesting to note that local thermodynamic equilibrium is established in the material due to the long life time of the sample, as compared to the laser-heated targets. A unique accelerator complex named, Facility for Antiprotons and Ion Research (FAIR), is under construction at Darmstadt. This is an international project that includes construction of a heavy ion synchrotron, SIS100, that will deliver intense particle beams of all stable species from protons up to uranium. High-Energy-Density (HED) physics is one of the fields of research that will be thoroughly studied at this facility. An international collaboration named HEDP@FAIR^10^, has been formed to oversee the construction of the experimental facilities and later to organize the running of the experiments. An interesting scientific proposal has been prepared for the HED physics experiments to be carried out by this collaboration. This experimental proposal has resulted from extensive theoretical studies over the past two decades, that include detailed numerical simulations and analytic modeling reported in numerous publications, see for example^11–34^. According to these studies, an ion beam can be employed to generate HED matter using two completely different schemes. In one case, states of high entropy and high pressure are generated in solid material by direct isochoric and uniform heating by the beam. Subsequent isentropic expansion of the heated material will allow one to access important HED states including an expanded hot liquid, two–phase liquid–gas state, critical parameters and strongly coupled plasmas. Such experiments named, HIHEX (Heavy Ion Heating and Expansion) will be done at FAIR to measure the equation-of-state (EOS) and transport properties of these different phases of HED matter.

In the second scheme, an intense ion beam with an annular focal spot is used to drive a multi-layered cylindrical target, that is comprised of a sample material enclosed in a heavy shell of a high-Z material. This beam–target set up generates a multiple shock reflection scheme that leads to a low-entropy compression of the sample material. Such a compression scheme produces extreme physical conditions that are expected to exist at planetary cores. This experimental proposal is named, LAPLAS (Laboratory Planetary Science). This experiment is designed to carry out planetary physics research. Another version of the LAPLAS scheme in which the target is driven by an ion beam with a circular focal spot, is also considered. In such a configuration, the sample material is not only compressed, but is directly heated by the beam as well. This generates a moderate entropy compression, which is used in the work presented in this paper, that is based on hydrodynamic simulations carried out using a 2D hydrodynamic code, BIG2^35^. Our simulations show that using the parameters of the SIS100 uranium beam, it is possible to produce macroscopic scale diamonds with mm^3^ dimensions.

**Figure 1:**
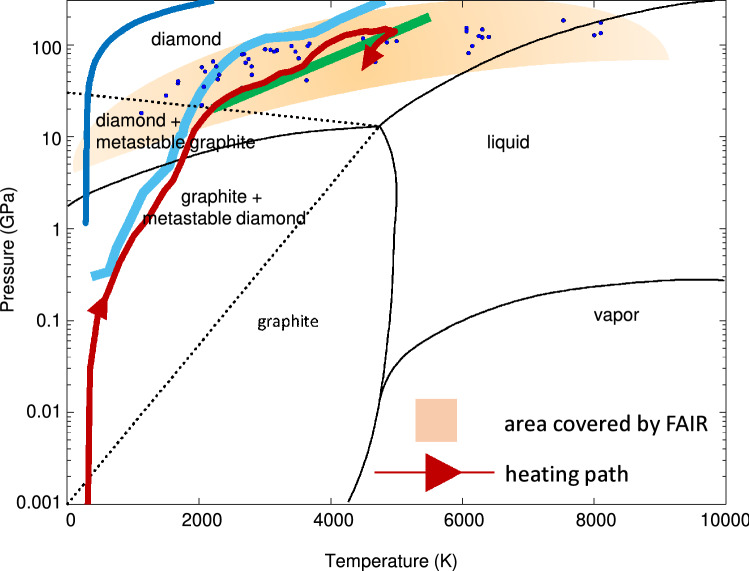
Carbon phase diagram (P–T)^36^, showing the area accessible using the FAIR facility. For comparison, the isentrope of Uranus (green line)^37^, the Earth geotherm (light blue line)^38^ and the compression path of a single shock (blue line) using the SESAME database, are included.

On Fig. 1, which is the carbon phase diagram^36^, we plot the simulated pressure–temperature values obtained in the carbon sample, reported in Tables 1, 2, 3, 4, 5, 6, 7, 8, 9, 10, 11, and 12, thus showing the region accessible using the FAIR facility. Each dot represents the pressure-temperature point achieved using a particular set of beam and target parameters. It is seen that almost all the points lie in the diamond part of the phase diagram. This means that over the wide range of the beam and the target parameters considered in this study, carbon sample can be transformed into diamond. The red solid line shows the time evolution of the temperature and pressure in the sample for a particular case in which the beam intensity is 3 × 10^11^ ions per bunch, the FWHM is 3 mm and the initial sample radius is 0.3 mm (Table 3, third row). This line shows the thermodynamic path followed by the sample in this case, and finally entering the diamond phase. For comparison, the isentrope of Uranus (green line)^37^, the Earth geotherm (light blue line)^38^ and the compression path of a single shock (blue line) obtained using the SESAME database, are included.

## Description of the FAIR accelerator facility

**Figure 2:**
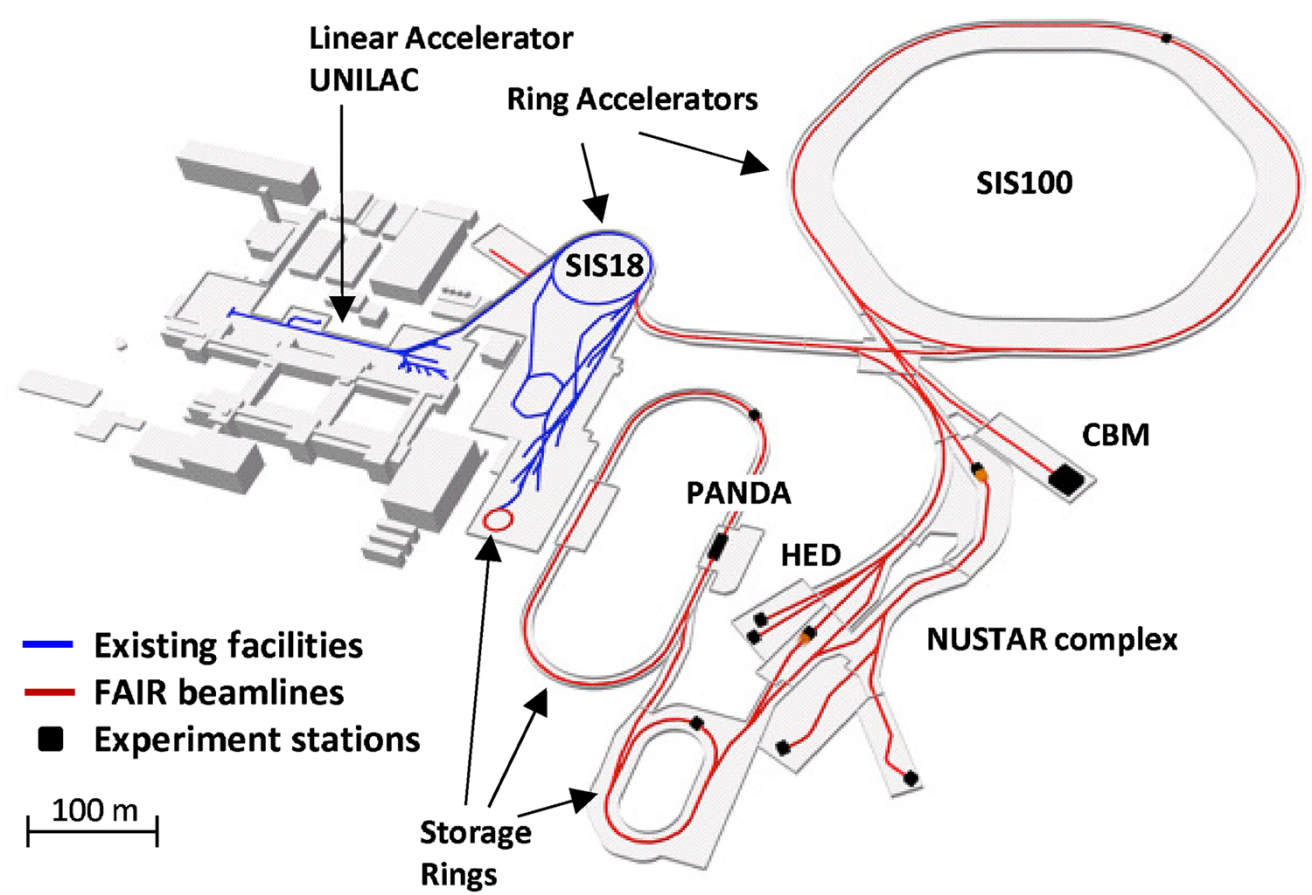
Overview of the FAIR (red) and GSI (blue) accelerator complex. The HED target station is located in a multi-purpose fully-shielded building: the APPA cave. *CBM* Compressed Baryonic Matter experiment, *NUSTAR* NUclear STructure Astrophysics and Reactions, *PANDA* antimatter research. *APPA* Atomic, Plasma Physics and Applications.

FAIR, currently under construction in Darmstadt, Germany, is a heavy-ion accelerator complex exploiting a synchrotron with a rigidity of 100 Tm (SIS100) capable of accelerating any ion from hydrogen to uranium to relativistic energies above 1 GeV/u. The FAIR synchrotron will be seeded by the accelerator facility of GSI and expands its capabilities greatly. The main purpose of this new accelerator is to re-create, in the laboratory, the extreme conditions that could be found in the universe from its creation to the complex nuclear processes taking place during stellar genesis and explosions^39^. To cover the breadth of applications foreseen at FAIR, the ring accelerator will be complemented by a series of storage rings targeting rare isotopes^40–42^ and anti-protons^43^, various target stations and a nuclear fragment separator, as depicted in Fig. 2.

For HED-related studies, FAIR will feed a dedicated ion beamline with heavy-ion beams with unprecedented intensities of up to 5 × 10^11^ with ion bunches as short as 70 to 200 ns^44^, whereas the heavy-ion beams with the highest intensities will be reached for low charge state (for example, U28^+^). To fully exploit this, a set of super-conducting magnets will focus the ion beam with low charge-to-mass ratio to sub-millimeter dimensions, only limited by the beam emittance. For the study presented here, we do not expect to need reaching this limit, as the smallest beam size considered, is still twice large as the emittance-limited beam. When taken collectively, the particles found in the intense heavy-ion pulses can be used to deposit large amounts of energy in excess of 100 kJ/cm^3^ over cubic-mm volumes uniformly in a short time, to reach high-energy-density states of matter in pressure and temperature. Compared to other methods used to reach HED states with shocks, isochoric heating alleviates the coupling between pressure and temperature found in a shock and enables exploring the full phase-space diagram of equation of states. On the other hand, the high pressure can also be used to produce a multiple-shock reflection scheme to generate planetary core conditions (the LAPLAS experiment). In addition, the strain rates found with such HED driver are lower than those of the more-commonly-used laser-driven shocks such that spatially uniform HED states in local thermodynamical equilibrium over cubic-mm samples can be obtained. For this reason, a dedicated target station in the multi-purpose APPA cave^45^ is being foreseen, where the LAPLAS setup will be installed.

In the summer of 2018, the civil construction of this new accelerator started next to the existing facility of GSI. Currently, the civil engineering work focuses on the experimental areas CBM, NUSTAR and APPA, after the synchrotron and connection tunnels have been successfully completed. A large fraction of the accelerator components have already been delivered and await installation in a nearby storage facility. In parallel, test and commissioning experiments using the upgraded GSI injector are taking place at the existing facilities, until the first caves become operational in the near future. As of today, a ramping of the FAIR performance to full specifications is expected in the second half of this decade.

## Method

In this section we provide the beam–target geometry of the proposed LAPLAS experimental scheme designed to generate a low-entropy compression of the sample material. We consider two different beam–target arrangements of the scheme that allow to access different parts of the phase diagram. In one case the target is driven by a hollow beam that has an annular focal spot, whereas in the other setup, the target is irradiated with a beam having a circular focal spot. In the following we describe in detail how these two different schemes work.

### Experimental set up using an annular focal spot

**Figure 3:**
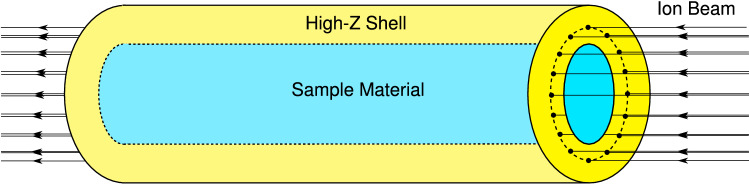
Beam–target set up of the LAPLAS scheme using an annular focal spot.

Figure 3 shows the beam–target geometry of this experimental scheme. A multi-layered cylindrical target is considered, that is comprised of a sample material, which is enclosed in a heavy shell of a high-Z material. An intense ion beam that has an annular (ring-shaped) focal spot, is incident on one face of the cylinder. The annular focal spot can be generated using an rf-wobbler that rotates the beam with very high frequency. Such a system is being designed for this experiment within the framework of the HEDP@FAIR collaboration. Detailed analysis of the energy deposition symmetry issues related to a wobbler have been analyzed and reported in^46^, while design of a prototype wobbler system has been reported in^47^.

**Figure 4:**
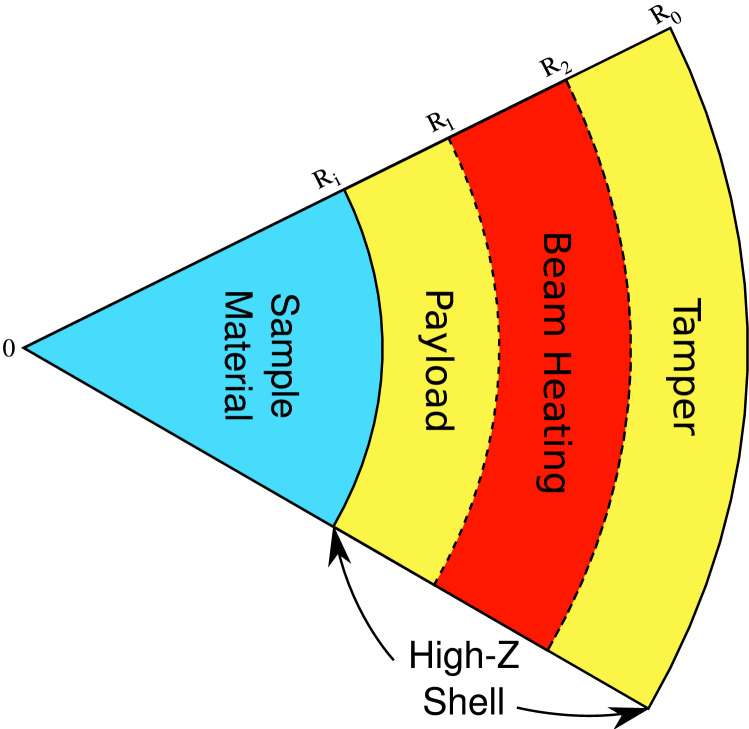
Cross sectional view of the LAPLAS target.

The inner radius of the annulus is considered to be larger than the radius of the sample material. This avoids strong direct heating of the sample by the ion beam as schematically shown in Fig. 4, which represents the cross sectional view of the LAPLAS target. Moreover, the outer radius of the focal spot ring should be smaller than the outer radius of the surrounding high-Z shell. Figure 4 shows that a layer of cold material from the high-Z shell known as “pusher” or “payload”, is created between the sample material and the beam-heated region. The payload plays an important role in placing the compression on the desired adiabat. It is also seen that a cold shell around the beam-heated zone acts as a tamper that confines the implosion for a longer time. It is also desirable that the target length is less than the range of the driver ions so that the Bragg peak does not lie inside the target that ensures uniform energy deposition in the longitudinal direction. The high level of energy deposition in the target, raises the temperature significantly that generates high pressure. This high pressure drives a shock inwards, along the radial direction. The shock enters the payload, and is subsequently transmitted into the sample, and then reflected at the cylinder axis. This reflected shock moves outwards along the radial direction and is re-reflected at the sample-shell boundary. This process is repeated a few times, while the boundary continues to move inwards, thereby compressing the sample slowly. The required sample physical conditions are achieved when the inward motion of the payload is stopped by the high pressure in the compressed sample. This scheme generates a low-entropy compression of the sample material that leads to the exotic physical conditions that are expected to exist in the planetary cores^21^.

### Experimental set up using a circular focal spot

**Figure 5:**
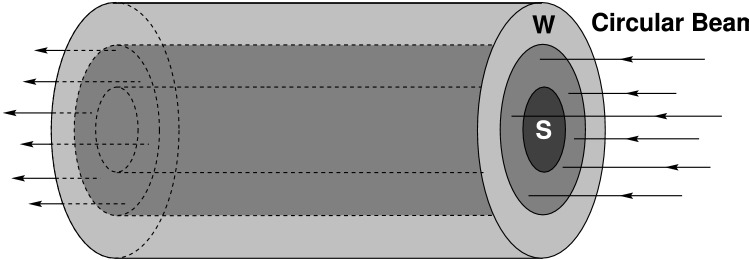
Beam–target set up of the LAPLAS scheme using a circular focal spot.

Figure 5 shows the beam–target setup of the experimental scheme that uses a beam with a circular focal spot. One face of the target is irradiated with the beam such that the beam axis coincides with the target axis. The ion range is larger than the target length so the energy deposition along the particle trajectory is uniform. In this configuration, the sample material is also directly heated by the ion beam, together with part of the surrounding high-Z shell that lies within the radius of the focal spot. In practice, the focal spot radius is considered to be equal to the full width at half maximum (FWHM) of the Gaussian distribution. This means that, unlike the other LAPLAS scheme, no high-density payload from the surrounding high-Z shell is formed around the sample. It is to be noted that the sample is preheated by the ion beam that leads to a higher pressure. However, the pressure in the surrounding heated part of the high-Z shell is much higher because of the large density difference in the two regions. This configuration therefore, also leads to a multiple shock reflection scheme, although the first shock is weaker compared to the other LAPLAS scheme due to the preheating (pre-pressure) in the sample. In fact these two different experimental schemes allow access to different parts of the phase diagram. The main advantage of the present scheme is that it works without employment of a wobbler.

## Beam and target parameters used in the study

In this study we consider the LAPLAS scheme using a circular focal spot with beam–target setup shown in Fig. 5. A uranium bunch with a particle energy of 2 GeV/u is used, while temporal intensity profile is parabolic with a duration of 200 ns (foot-to-foot). The radial intensity distribution in the focal spot is assumed to be Gaussian. Different values of the FWHM including, 2, 2.5, 3, 3.5 and 4 mm, respectively, are considered. For practical purposes, the FWHM of the distribution is assumed to be the beam radius. Such large sizes of the focal spot means that the focusing requirements of the beam are very relaxed. Moreover, different bunch intensities, for example, 5 × 10^10^, 10^11^, 2 × 10^11^ and 3 × 10^11^ ions, respectively, are used.

The target is comprised of a carbon sample that has a density of 2.25 g/cm^3^ and that is enclosed in a cylindrical shell of tungsten. Different values of the initial sample radius, R$_{si}$, including 200, 300, 400 and 500 μm, respectively, are considered. These lead to sample mass of 2.3, 6.4, 11.3 and 17.7 mg/cm, respectively. The outer radius of the tungsten shell is 5 mm. We also used numerous values of the cylinder length, including, 3, 4, 5, 6 and 7 mm, respectively.

## Description of the computer code used in the simulations

Computer code BIG2 is a two-dimensional hydrodynamic simulation model that is equipped with the necessary physics relevant to the problem of beam-matter heating and compression. The code can handle plane as well as cylindrical geometry.

A cold stopping model described in^48^, is used to calculate the ion beam energy deposition in the target. This approximation is valid, as in ion-beam heated targets, the temperature is rather low (below 10 eV) so that the ionization effects are negligible. The plasma effects on the stopping power become important when the ionization level in the material is significant, which requires a much higher temperature.

A semi-empirical EOS model described in^49,50^, is used to treat different phases of the target material. This model considers macroscopically-correct equation of state that accounts for solid, liquid and gaseous states as well as the melting and the evaporating two-phase regions. Due to the relatively long timescales (around 100 ns) involved in this problem, the target material is considered to be under conditions of the local thermodynamic equilibrium. In such a case one can apply in numerical modeling of expanded target the equation of state in tabular form, using Maxwell construction in the two-phase liquid-gas region, as it has been discussed in^34^.

The mechanical (elastic–plastic) properties of solid materials are taken into account using the non-linear Prandtl–Reuss model with the von Mises yield criterion, which is given by the following differential equations for the deviatoric part ${\textbf {S}}$ of the stress tensor $\mathbf{\sigma }= -P \ {\textbf {I}} + {\textbf {S}}$ (where *P* is the pressure, and ${\textbf {I}}$ is the identity tensor) ^51^:

1 $$\begin{aligned} \dot{{\textbf {S}}} = 2 G {\textbf {D}} \ \ \end{aligned}$$

If $${\textbf {S}}.{\textbf {D}} < 0$$ or $${\textbf {S}}. {\textbf {S}} < \frac{2}{3} Y^2$$

2 $$\begin{aligned} \dot{{\textbf {S}}} + 2 G {\textbf {S}} \frac{{\textbf {S}}.{\textbf {D}}}{{\textbf {S}}.{\textbf {S}}} = 2 G {\textbf {D}} \ \ \ \ \end{aligned}$$

If $${\textbf {S}}.{\textbf {D}} > 0$$ and $${\textbf {S}}. {\textbf {S}} = \frac{2}{3} Y^2$$.

Here *G* is the shear modulus, *Y* is the yield strength and both parameters are characteristic of the solid material, which to the scope of a parametric study are taken as independent and constant parameters. Besides, in Eqs. (1) and (2), ${\textbf {D}}$ is the strain rate tensor:

3 $$\begin{aligned} {\textbf {D}}=\frac{1}{2}\left( {\nabla }{} {\textbf {v}} + {\nabla }{} {\textbf {v}}^{T} \right) \ , \end{aligned}$$

where ${\textbf {v}}$ is the velocity field and the superscript *T* indicates the transpose tensor.

**Figure 6:**
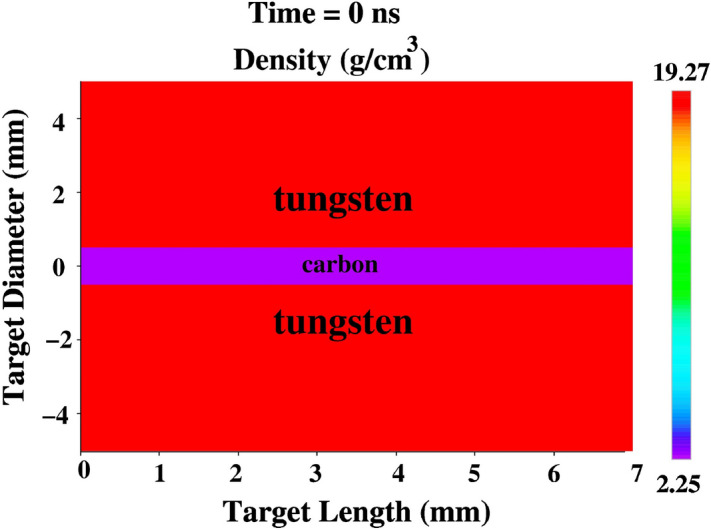
Initial target density distribution generated by BIG2 code.

The numerical algorithm of BIG2 is based on a Godunov type scheme^52^, which uses a finite-volume approach in the space-time domain. The fluxes are calculated using the solution of Riemann problem at each inter-cell boundary. It is a conservative scheme that has a second order accuracy in space and first order accuracy in time. The code is based on an Eulerian numerical scheme that uses curvilinear rectangular moving grid. The grid is adaptive to gradient of physical parameters (pressure, temperature, density) due to the condensation of the grid lines. The movement of the grid boundary is calculated in accordance with the type of the boundary, for example, it could be a shock front, a material interface, a free boundary, a rigid wall and so on. Reconstruction of the grid at the new time step is carried out by quasi-conformal mapping of the rectangular grid to the area with the numerical grid boundary calculated at the new time step, taking into account the gradient of the specified physical parameter. Further details about the numerical techniques used in the code can be found in reference^35^. The BIG2 code can treat multi-layered targets comprised of different materials and can handle complicated target geometries.

## Numerical simulations results

In this section we present the numerical simulation results obtained using the 2D hydrodynamic code BIG2. The beam and the target parameters used in this study are noted above. A semi-empirical EOS model^49,50^ is used for tungsten, while for carbon, the SESAME data^53^ is considered. The ion energy deposition is calculated using the SRIM computer code described in^48^. It is to be noted that on the time scale of our interest, thermal conduction is not important and therefore we exclude it in the simulations. We note that although the SIS100 is designed to deliver an intensity of 5 × 10^11^ uranium ions, our desired phase transition in carbon can be achieved using lower intensities.

### Bunch intensity of 3 × 10^11^

We assume a bunch intensity of 3 × 10^11^ uranium ions with a particle energy of 2 GeV/u. The temporal power profile is parabolic with a bunch length of 200 ns (foot-to-foot). The focal spot has circular geometry, that has a Gaussian intensity distribution in the radial direction, with a FWHM of 3 mm. The sample radius is 0.5 mm and the outer target radius is 5 mm, whereas the target length is 7 mm. The initial carbon and tungsten densities used in these simulations are 2.25 and 19.27 g/cm^3^, respectively. The target initial conditions are shown in Fig. 6, where we present the target density distribution generated by the BIG2 code at t = 0 ns.

**Figure 7:**
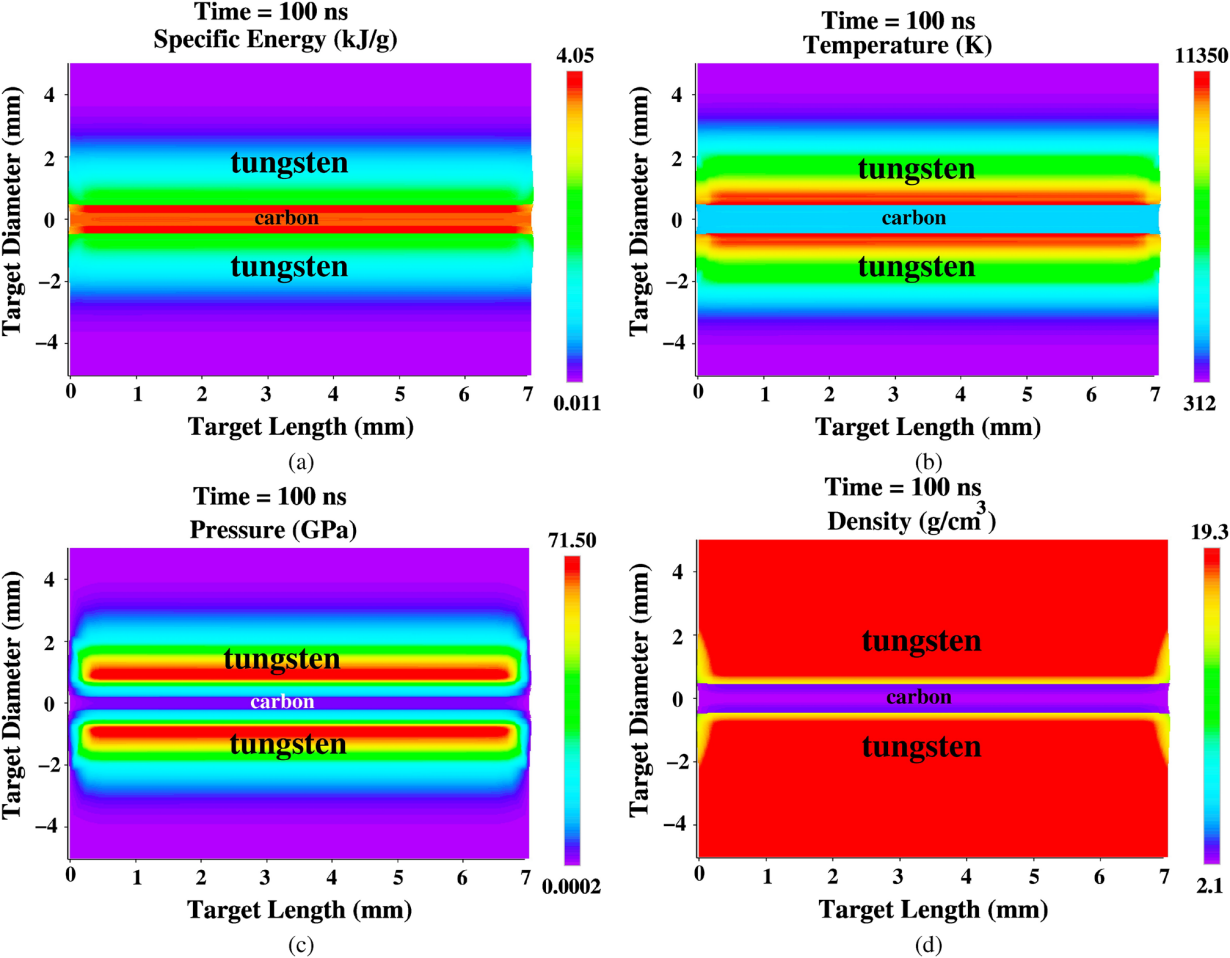
Target physical conditions generated by the BIG2 code at t = 100 ns, sample radius R$_{si}$ = 0.5 mm, outer target radius, R$_o$ = 5 mm, target length = 7 mm, bunch intensity = 3 × 10^11^ uranium ions, bunch length = 200 ns, ion energy = 2 GeV/u, (**a**) specific energy, (**b**) temperature, (**c**) pressure and (**d**) density.

The target physical conditions calculated by the BIG2 code at t = 100 ns (middle of the bunch), are presented in Fig. 7. It is seen in Fig. 7a, where we plot the specific energy distribution, that the specific energy in the carbon is about 4 kJ/g, which up to this time is purely due the beam heating.

The corresponding temperature distribution is given in Fig. 7b, that shows that the maximum temperature in the tungsten around the sample is of the order of 11,000 K. It is interesting to note that carbon has a higher value of specific energy compared to tungsten, whereas the temperature shows an opposite behavior.

The high temperature in the beam heated zone leads to high pressure and this pressure distribution is plotted in Fig. 7c. It is seen that tungsten region around the carbon sample has a high pressure of around 70 GPa, that drives a cylindrically converging shock in the radial direction.

The target density distribution at t = 100 ns is presented in Fig. 7d. It is seen that there is no compression in the tungsten region around the carbon because that region is strongly heated by the beam. Therefore no high density “payload” ring of tungsten is generated around the carbon.

**Figure 8:**
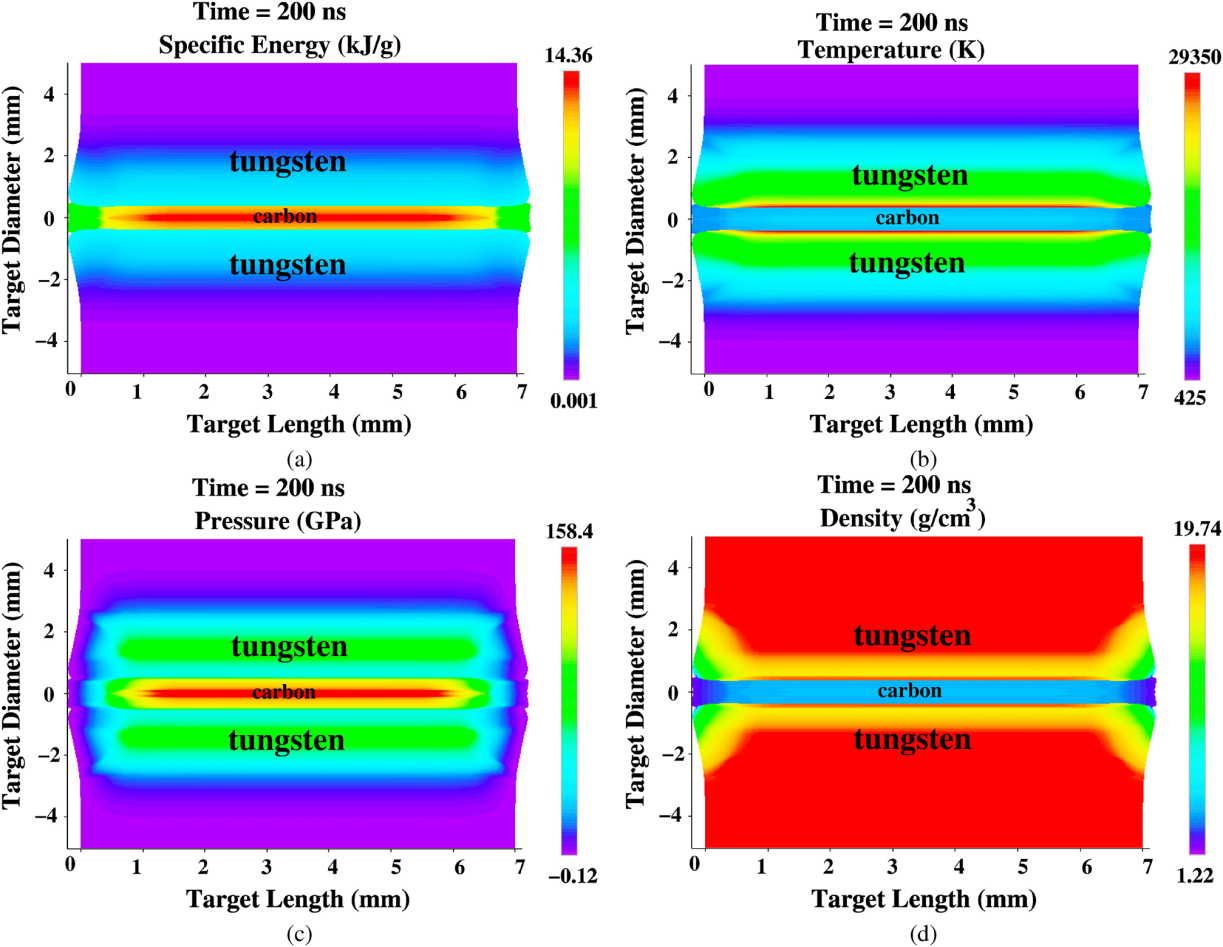
Same as in Fig. 6, but at t = 200 ns (end of the ion bunch).

The distributions of the target physical conditions generated by the BIG2 code at t = 200 ns (end of the bunch), are presented in Fig. 8. It is seen in Fig. 8a that by this time,the specific energy in the carbon is of the order of 14 kJ/g, which is a combined result of the direct beam heating and compressional heating.

In Fig. 8b, we present the corresponding temperature distribution, which shows that the maximum temperature in the tungsten around the sample is about 29,000 K, that decreases along the radius in the outward direction. This behavior is due to the Gaussian distribution of the intensity in the focal spot.

**Figure 9:**
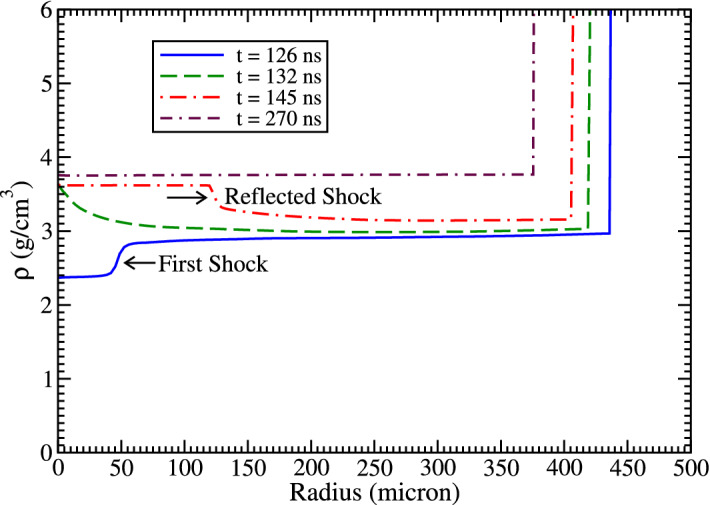
Density vs radius at different times at the middle of the axis.

Figure 8c shows that there is a high pressure of the order of 158 GPa in a region around the axis, while it has a lower value in the surrounding part. This is because the shock transmitted from the high pressure tungsten region is still reverberating between the axis and the tungsten–carbon boundary. This point will be explained in detail in Fig. 9.

The corresponding density distribution is shown in Fig. 8d.

In order to explain the details of the implosion process involved in this scheme, we plot in Fig. 9, the density vs radius at the middle of the axis (L = 3.5 mm), at different times during the implosion. It is seen that at t = 126 ns, the shock transmitted from the high pressure tungsten into carbon has arrived at a radial position of r = 50 μm. It is to be noted that due to the preheat of the unshocked material, the pressure ahead of the shock continuously increases, that weakens the shock. The density profile labelled with t = 132 ns shows that the shock has arrived at the axis, where it is reflected and the profile at t = 145 ns shows the position of the reflected shock at that time. This process is repeated a few more times, while the shock strength continuously decreases. The final compression is achieved at t = 270 ns, that shows a uniform density of the order of 3.75 g/cm^3^ along the entire radius. The vertical lines represent the carbon–tungsten boundary position at different times. It is seen that this boundary continuously moves inwards, thereby slowly compressing the sample material.

**Figure 10:**
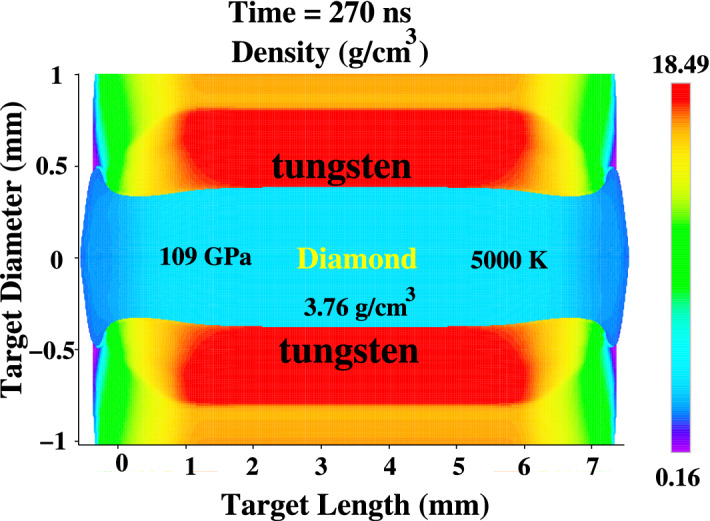
Achieved physical conditions at t = 270 ns.

**Figure 11:**
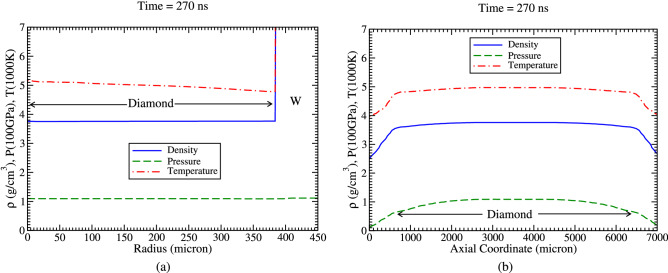
Achieved density, temperature and pressure profiles at t = 270 ns, (**a**) along radius at the middle of the axis and (**b**) along the axis (r = 0).

In Fig. 10, we present the physical conditions achieved at t = 270 ns. To increase the resolution of the results, we show only the inner 1 mm radius of the cylinder. It is seen that the density is about 3.75 g/cm^3^, the pressure is around 109 GPa, whereas the temperature is of the order of 5000 K. The carbon phase diagram (Fig. 1) shows that under these conditions, the carbon undergoes a phase transition and becomes diamond.

In order to have a better quantitative understanding of the results, it is useful to plot one-dimensional density, temperature and pressure profiles along the radius as well as along the axis, respectively. Profiles along the radius are plotted at the middle of the axis (L = 3.5 mm).

Figure 11a shows that the density, temperature and pressure is very uniform along the radius, which indicates that the problem can be treated with a one-dimensional hydrodynamic model along the radius. Therefore the rest of the results presented in this paper are based on one-dimensional version of the BIG2 code.

It is shown in Fig. 11b that the profiles are also uniform along the major part of the axis in the inner part of the sample. It is concluded from Fig. 11a and b that a cylindrical diamond with a length of about 6 mm and a radius of around 385 μm will be produced.

**Figure 12:**
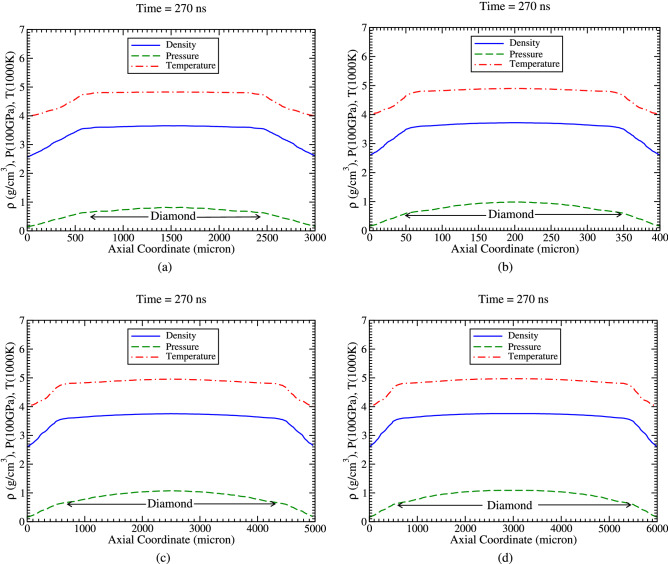
Density, temperature and pressure along axis at t = 270 ns, (**a**) target length = 3 mm, (**b**) target length = 4 mm, (**c**) target length = 5 mm and (**d**) target length = 6 mm.

It is also to be noted that optimization of the target length is an important issue, as a shorter target is desirable for the facilitation of the diagnostics. We therefore have carried out simulations using shorter targets with length, L = 3, 4, 5 and 6 mm, respectively. Rest of the parameters are the same. The results are plotted in Fig. 12.

In Fig. 12a, we plot the profiles of the achieved physical parameters vs axis at t  = 270 ns, using a cylinder length of 3 mm. It is seen that the achieved values of the parameters are the same as before, but the length of the diamond is about 1.7 mm.

The results corresponding to L = 4 mm are presented in Fig. 12b. It is seen that the length over which the physical conditions are uniform is about 3 mm, which represents the length of the produced diamond.

Similarly, in Fig. 12c we plot the density, temperature and pressure profiles along the axis at t = 270 ns for the case, L = 5 mm, which shows that the length of the produced diamond is about 4 mm.

The respective physical conditions obtained using L = 6 mm, are presented in Fig. 12d, that shows that the produced diamond has a length of about 5 mm.

The radius of the diamond zone is the same (about 385 μm) in all the above cases.

**Table 1:** Achieved physical conditions in compressed carbon using bunch intensity = 3 × 10^11^ uranium ions, bunch length = 200 ns, particle energy = 2 GeV/u, initial sample radius, R$_{si}$ = 500 μm, sample mass = 17.7 mg/cm.

No.	FWHM (mm)	$\rho$ (g/cm^3^	P (GPa)	T (K)	t$_{impl}$ (ns)	State
1	2.0	3.67	127	8000	240	Diamond
2	2.5	3.74	120	6300	255	Diamond
3	3.0	3.76	109	5000	270	Diamond
4	3.5	3.75	98	3900	288	Diamond
5	4.0	3.74	87	3180	308	Diamond

**Table 2:** Achieved physical conditions in compressed carbon using bunch intensity = 3 × 10^11^ uranium ions, bunch length = 200 ns, particle energy = 2 GeV/u, initial sample radius, R$_{si}$ = 400 μm, sample mass = 11.3 mg/cm.

No.	FWHM (mm)	$\rho$ (g/cm^3^	P (GPa)	T (K)	t$_{impl}$ (ns)	State
1	2.0	3.70	134	8100	231	Diamond
2	2.5	3.75	122	6400	246	Diamond
3	3.0	3.75	106	4850	264	Diamond
4	3.5	3.73	93	3830	285	Diamond
5	4.0	3.72	84	3140	297	Diamond

**Table 3:** Achieved physical conditions in compressed carbon using bunch intensity = 3 × 10^11^ uranium ions, bunch length = 200 ns, particle energy = 2 GeV/u, initial sample radius, R$_{si}$ = 300 μm, sample mass = 6.4 mg/cm.

No.	FWHM (mm)	$\rho$ (g/cm^3^	P (GPa)	T (K)	t$_{impl}$ (ns)	State
1	2.0	3.85	172	8100	192	Diamond
2	2.5	3.86	146	6300	205	Diamond
3	3.0	3.82	123	4840	220	Diamond
4	3.5	3.80	104	3970	215	Diamond
5	4.0	3.75	88	3080	261	Diamond

**Table 4:** Achieved physical conditions in compressed carbon using bunch intensity = 3 × 10^11^ uranium ions, bunch length = 200 ns, particle energy = 2 GeV/u, initial sample radius, R$_{si}$ = 200 μm, sample mass = 2.8 mg/cm.

No.	FWHM (mm)	$\rho$ (g/cm^3^	P (GPa)	T (K)	t$_{impl}$ (ns)	State
1	2.0	3.92	183	7530	160	Diamond
2	2.5	3.90	151	6060	179	Diamond
3	3.0	3.83	123	4700	200	Diamond
4	3.5	3.81	103	3670	215	Diamond
5	4.0	3.75	89	3000	230	Diamond

In Table 1 we present the precise values of the achieved physical parameters obtained in the simulations using different values of the focal spot size represented by the FWHM of the Gaussian intensity distribution, including, 2, 2.5, 3, 3.5 and 4 mm, respectively. It is seen that using FWHM = 2 mm, the achieved density is about 3.67 g/cm^3^, the achieved pressure is around 127 GPa, whereas the temperature is 8000 K in the compressed carbon sample, while the implosion time, t$_{impl}$, is 240 ns. The second row of this table shows that using FWHM = 2.5 mm, the corresponding achieved physical parameters in the compressed carbon have values, 3.74 g/cm^3^, 120 GPa and 6300 K, respectively. The implosion time in this case is 255 ns. The table further shows that with the increasing value of the FWHM, the final pressure and the temperature achieved in the sample decrease correspondingly. The final density, on the other hand, remains rather insensitive. This is because the hydrodynamic processes including the multiple-shock reflection remain unchanged. Moreover, the implosion time increases as the FWHM increases. This behavior is very logical because the level of the specific energy deposition decreases as the focal spot size is increased, that leads to reduction in the temperature and pressure, which makes the implosion slower and hence t$_{impl}$ becomes longer. It is important to note that according to the carbon phase diagram shown in Fig. 1, all the sets of the physical conditions noted in the above table correspond to the diamond phase. In the present case, the sample mass is is 17.7 mg/cm, whereas, the final sample radius at the time of achieved compression is about 385 μm.

In Table 2, we present results using a smaller mass of the sample, namely, 11.3 mg/cm, that corresponds to an initial sample radius, R$_{si}$ = 400 μm, while rest of the parameters are the same as in the previous case. This table shows that using FWHM = 2 mm, the density, pressure and temperature achieved in the compressed sample are 3.7 g/cm^3^, 134 GPa and 8100 K, respectively, whereas the implosion time, t$_{impl}$ is 231 ns. Comparing these values with the corresponding values in Table 1, it is seen that the values of the physical parameters are similar, while the implosion time in the present case is somewhat shorter. The same behavior is seen for the other values of the FWHM. The results are therefore insensitive to a significant variation in the sample mass. We note that the final radius of the sample at the achieved compression is about 305 μm.

In Table 3, we present results considering an initial sample radius, R$_{si}$ = 300 μm that corresponds to a sample mass of 6.36 mg/cm. Rest of the parameters are the same as in the previous two cases. This table shows that using FWHM of 2 mm, the maximum sample density is 3.85 g/cm$^3$, which is higher than the corresponding values in the previous two cases noted in Tables 1 and 2, whereas the final temperature is the same (8100 K). This is because in the present case, the sample mass is significantly smaller, while the driving input energy is the same. Consequently, the final pressure in the present case is higher (172 GPa) due to a higher density. Moreover, the implosion time, t$_{impl}$ is 192 ns, which is shorter than the corresponding values in the previous cases. A similar pattern is seen in the results presented in the following rows representing different values of the spot size. The achieved sample radius at the time of maximum compression is about 230 μm.

Finally, in Table 4 we present the results using the shortest value of R$_{si}$ = 200 μm, which corresponds to a sample mass of  2.8 mg/cm. It is seen that using FWHM of 2 mm, t$_{impl}$ is 160 ns, which means that a significant part of the beam energy is not available for heating, and as a consequence the maximum sample temperature is 7530 K, which is less than the corresponding values in the previous three tables. However, in the present case, the sample mass is much smaller and is therefore compressed more efficiently. This leads to a higher density of 3.92 g/cm^3^, which results in a higher pressure of 183 GPa. In case of FWHM of 3 mm and above, t$_{impl}$ is 200 ns and above, which means that the entire bunch energy is available for heating. A comparison of the results with the corresponding parameters in the three previous tables show that the values are comparable. The final radius of the compressed sample is 153 μm.

### Bunch intensity of 2 × 10^11^

In the following we summarize the results obtained using a lower bunch intensity of 2 × 10^11^ ions, while rest of the beam and the target parameters are the same as used in the different cases discussed previously. In Table 5 we present the precise values of the physical parameters obtained using various beam spot sizes characterized by different values of the FWHM of the Gaussian intensity distribution. In the present case, the sample mass is 17.7 mg/cm.

**Table 5:** Achieved physical conditions in compressed carbon using bunch intensity = 2 × 10^11^ uranium ions, bunch length = 200 ns, particle energy = 2 GeV/u, initial sample radius, R$_{si}$ = 500 μm, sample mass = 17.7 mg/cm.

No.	FWHM (mm)	$\rho$ (g/cm^3^	P (GPa)	T (K)	t$_{impl}$ (ns)	State
1	2.0	3.55	81	6090	282	Diamond
2	2.5	3.58	65	4675	328	Diamond
3	3.0	3.57	54	3530	376	Diamond
4	3.5	3.56	47	2800	414	Diamond
5	4.0	3.53	42	2280	450	Diamond

It is seen in Table 5, that the achieved values of the physical parameters are lower than their corresponding values presented in Table 1. This is because a lower beam intensity means lower specific energy deposition, that leads to a lower temperature, which translates into a lower driving pressure, that produces a lower compression. Moreover, t$_{impl}$ is also longer than the corresponding values noted in Table 1. However, Fig. 1 shows that all the sets of the physical conditions achieved in the present case belong to the diamond phase of carbon. The final radius of the compressed carbon sample is about 395 μm.

Results obtained using this bunch intensity, considering a smaller sample mass of 11.3 mg/cm, are noted in Table 6. The corresponding results using the previous higher intensity are presented in Table 2. It is seen that achieved carbon density, temperature and pressure values shown in Table 6 are significantly lower than their corresponding values given in Table 2. However, the different sets of the physical parameters noted in Table 6 indicate that the sample will be transformed into the diamond phase, as shown in Fig. 1. It is also interesting to note that a comparison between Tables 5 and 6 (that use the same bunch intensity of 2 × 10^11^ ions), shows that despite the difference in the sample mass, the results are quite similar, that indicates the robustness of this scheme. The final radius of the compressed carbon sample is about 315 μm.

**Table 6:** Achieved physical conditions in compressed carbon using bunch intensity = 2 × 10^11^ uranium ions, bunch length = 200 ns, particle energy = 2 GeV/u, initial sample radius, R$_{si}$ = 400 μm, sample mass = 11.3 mg/cm.

No.	FWHM (mm)	$\rho$ (g/cm^3^	P (GPa)	T (K)	t$_{impl}$ (ns)	State
1	2.0	3.65	97	6150	249	Diamond
2	2.5	3.67	85	4630	273	Diamond
3	3.0	3.66	71	3510	305	Diamond
4	3.5	3.63	58	2795	345	Diamond
5	4.0	3.60	47	2300	387	Diamond

**Table 7:** Achieved physical conditions in compressed carbon using bunch intensity = 2 × 10^11^ uranium ions, bunch length = 200 ns, particle energy = 2 GeV/u, initial sample radius, R$_{si}$ = 300 μm, sample mass = 6.4 mg/cm.

No.	FWHM (mm)	$\rho$ (g/cm^3^	P (GPa)	T (K)	t$_{impl}$ (ns)	State
1	2.0	3.76	124	6250	210	Diamond
2	2.5	3.75	104	4580	230	Diamond
3	3.0	3.71	84	3460	258	Diamond
4	3.5	3.68	70	2750	285	Diamond
5	4.0	3.64	58	2260	320	Diamond

Next, we present in Table 7 the simulation results obtained using a sample mass of 6.4 mg/cm. The corresponding results that are produced using a higher bunch intensity are given in Table 3. A comparison between these two tables shows that same pattern exists as before. The values of the physical parameters noted in Table 7 are lower than those given in Table 3 due to the reduction in the bunch intensity. Again, it is seen that all the different sets of the physical conditions correspond to the diamond phase of carbon, as shown in Fig. 1.

**Table 8:** Achieved physical conditions in compressed carbon using bunch intensity = 2 × 10^11^ uranium ions, bunch length = 200 ns, particle energy = 2 GeV/u, initial sample radius, R$_{si}$ = 200 μm, sample mass = 2.8 mg/cm.

No.	FWHM (mm)	$\rho$ (g/cm^3^	P (GPa)	T (K)	t$_{impl}$ (ns)	State
1	2.0	3.85	141	6060	170	Diamond
2	2.5	3.81	117	4490	186	Diamond
3	3.0	3.76	97	3400	205	Diamond
4	3.5	3.72	79	2680	230	Diamond
5	4.0	3.68	66	2210	255	Diamond

The simulation results obtained using the smallest sample mass of 2.8 mg/cm, are presented in Table 8. These results again indicate that the sets of values of physical parameters correspond to the diamond phase of carbon. It is thus concluded that using this bunch intensity, it is possible to transform carbon into diamond considering a wide range of beam and target parameter space.

### Bunch intensity of 10^11^

In the following we present a summary of the results obtained using a bunch intensity of 10^11^ ions. We note that only the lower values of the sample mass, namely, 2.8 and 6.4 mg/cm, are considered. The implosion becomes inefficient with the larger sample mass because the driving energy is not sufficient. For the same reason we do not consider the FWHM of 4 mm, because the focal spot is too large to provide enough specific energy to efficiently drive the implosion.

In Table 9, we present the achieved values of the physical parameters obtained using a sample mass of 6.4 mg/cm. It is seen that the density is around 3.6 g/cm^3^ for all the four different values of the FWHM, whereas, the temperature and the pressure, respectively are reduced as the FWHM increases. The implosion time also increases which indicates that the implosion becomes slower as the spot size is increased. The reason for this behavior is explained in the previous sections. It is also to be noted that the values of the physical parameters noted in Table 9, suggest that the diamond phase of carbon can be achieved with these beam and target parameters.

**Table 9:** Achieved physical conditions in compressed carbon using bunch intensity = 10^11^ uranium ions, bunch length = 200 ns, particle energy = 2 GeV/u, initial sample radius, R$_{si}$ = 300 μm, sample mass = 6.4 mg/cm.

No.	FWHM (mm)	$\rho$ (g/cm^3^	P (GPa)	T (K)	t$_{impl}$ (ns)	State
1	2.0	3.66	78	3755	235	Diamond
2	2.5	3.66	64	2705	261	Diamond
3	3.0	3.62	51	2090	291	Diamond
4	3.5	3.53	38	1675	347	Diamond

**Table 10:** Achieved physical conditions in compressed carbon using bunch intensity = 10^11^ uranium ions, bunch length = 200 ns, particle energy = 2 GeV/u, initial sample radius, R$_{si}$ = 200 μm, sample mass = 2.8 mg/cm.

No	FWHM (mm)	$\rho$ (g/cm^3^	P (GPa)	T (K)	t$_{impl}$ (ns)	State
1	2.0	3.76	97	3630	178	Diamond
2	2.5	3.71	78	2650	203	Diamond
3	3.0	3.65	57	2040	243	Diamond
4	3.5	3.58	40	1680	294	Diamond

Table 10 presents the simulation results using a sample mass of 2.8 mg/cm. It is seen that using a FWHM of 2 mm, a density of 3.76 g/cm^3^, whereas in the previous case noted in Table 9, it is 3.66 g/cm^3^. The pressure in the present case is 97 GPa compared to a lower value of 78 GPa in Table 9. The temperature, on the hand is comparable in the two cases. This is due to the fact that the driving input power is the same in both cases, so the smaller mass is compressed more efficiently, that leads to a higher density.

It is also interesting to note that for the higher values of the FWHM, namely, 3 and 3.5 mm, the values corresponding of the parameters are similar in the two tables. This is because for such large focal spot size the specific energy deposition is low and the sample material is not compressed efficiently, even for the smaller mass.

### Bunch intensity of 5 × 10^10^

Simulations have also been done using a bunch intensity of 5 × 10^10^ and the results are presented below. We note that due to the low beam intensity, smaller values of the FWHM are considered to keep the specific energy deposition level reasonable. Moreover, smaller sample mass is considered to make the implosion efficient.

In Table 11 we present the values of the physical parameters achieved using a sample mass of 6.4 mg/cm. It is seen that according to Fig. 1, these values correspond to the diamond phase of carbon. Table 12 shows the same parameters as Table 11, but using a sample mass of 2.8 g/cm. It is seen that in this case, the diamond phase can also be achieved.

**Table 11:** Achieved physical conditions in compressed carbon using bunch intensity = 5 × 10^10^ uranium ions, bunch length = 200 ns, particle energy = 2 GeV/u, initial sample radius, R$_{si}$ = 300 μm, sample mass = 6.4 mg/cm.

No	FWHM (mm)	$\rho$ (g/cm^3^	P (GPa)	T (K)	t$_{impl}$ (ns)	State
1	2.0	3.27	35	2070	332	Diamond
2	2.5	3.12	28	1495	340	Diamond
3	3.0	2.82	18	1125	368	Diamond

**Table 12:** Achieved physical conditions in compressed carbon using bunch intensity = 5 × 10^10^ uranium ions, bunch length = 200 ns, particle energy = 2 GeV/u, initial sample radius, R$_{si}$ = 200 μm, sample mass = 2.8 mg/cm.

No	FWHM (mm)	$\rho$ (g/cm^3^	P (GPa)	T (K)	t$_{impl}$ (ns)	State
1	2.0	3.5	41	3630	178	Diamond
2	2.5	3.4	34	2650	203	Diamond
3	3.0	2.9	22	2040	243	Diamond

It is interesting to note that the estimated peak pressures of the Canyon Diablo and Popigai are of the order of 60–100 GPa, respectively. Our simulations suggest that such huge pressures can be generated in the LAPLAS experiments. These experiments, therefore, will not only improve our knowledge regarding the structure and evolution of the carbon rich planets, but will also be useful in understanding the formation processes of the carbon structures found at such sites.

## Diagnostics

The extensive hydrodynamic calculations of LAPLAS implosions described in the previous sections suggest that this scheme allows to dynamically compress carbon samples to conditions where formation of cubic diamond or even more exotic structures, as found at meteor impact sites^54^ are expected. Consequently, it is of high interest to recover the materials formed in this transient state. Post-mortem analysis of recovered samples would allow to use the wide range of materials characterization techniques (e.g. Raman spectroscopy, X-ray diffraction, electron microscopy, Auger electron spectroscopy) in order to find, identify and quantify the new materials generated^55^. This will enable a better understanding of the formation processes, possibly finding so far unknown novel phases, or even make them available for applications. While in-situ X-ray diffraction of laser-shock compressed samples has shown the formation of nano-diamonds, and even indicate that these survive the rapid (few nanoseconds) decompression to ambient densities^8^, their successful recovery remains a great challenge^55^. After shock-breakout the samples are ejected in the course of a free-surface release with velocities up to 20 km/s. Hyper-velocity impacts upon slowing down of the ejecta in a suitable catcher can destroy the new phases, and the elusive material is dispersed over a large area. The cylindrical compression in the LAPLAS scheme has the enormous advantage that the high-pressure sample is generated essentially at rest. Also, both the compression and decompression phase are significantly longer, extending over hundreds of nanoseconds, potentially resulting in a higher yield and larger crystal sizes. Finally, the large volumes (> mm^3^) considered for these experiments will significantly ease the recovery of sample material.

Besides such post-mortem analysis, in-situ monitoring of the hydrodynamic performance of the implosion and the conditions achieved upon stagnation will be crucial. This would allow to test and benchmark the hydrodynamic calculations, which is of particular importance as both the sample and the pusher traverse a wide parameter range in the strongly coupled partly degenerate regime (so-called “warm dense matter”), where the equation-of-state is poorly known. Here, we propose X-ray radiography, enabled by powerful X-ray sources driven by a high-energy laser system that is being planned for plasma physics experiments at FAIR. X-ray radiography using intense bursts of X-rays from laser-produced plasmas is an indispensable diagnostic tool in HED experiments, as it allows to measure the density distribution of the rapidly evolving dense samples. For example, in shock experiments measurement of shock velocity and compression ratio gives access to EOS at extreme pressures on the Hugoniot^56^. In inertial confinement fusion the implosion velocity, symmetry and remaining mass of the fuel shell provides crucial feedback for tuning the implosion performance^57^.

**Figure 13:**
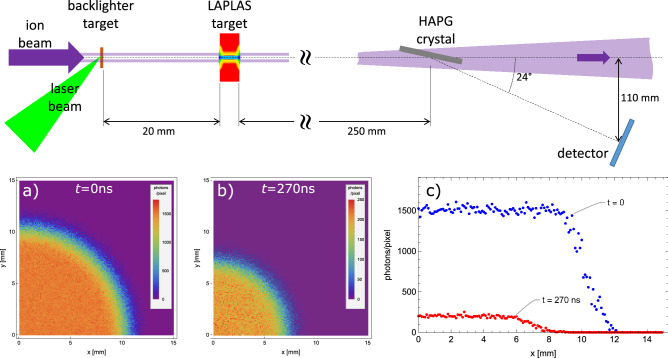
(top) Schematic of the proposed set up for on-axis radiographic imaging using a laser-driven X-ray source, and (bottom) simulated radiographic images of (**a**) the initial target, (**b**) after compression at a time of 270 ns, and (**c**) central lineouts at both probe times.

In the past, we have proposed high-intensity laser-driven hard X-ray radiography to monitor the implosion and strong compression in the LAPLAS scheme^21^. Laser pulses focused to relativistic intensities ($\ge$ 10^18^ W/cm^2^) are well known to produce copious amounts of supra-thermal electrons, which in turn excite bremsstrahlung with photon energies well above 100 keV^58^, and source sizes down to 5 μm, limited only by the target dimensions^59^. While a high-energy high-intensity (kJ pulse energy in picosecond duration) laser is foreseen for HED experiments at FAIR to drive such hard X-ray sources, for the initial phase of the experimental program a lower energy long-pulse (nanosecond) laser will be available. Focused to intensities of order 10^15^ W/cm^2^, a plasma with few keV temperature is produced, expanding on nanosecond timescales to approximately 100 μm. Collisionally excited resonance line emission from highly charged ions in this plasma can reach appreciable rates. The conversion efficiency of laser energy into to Helium-alpha X-ray line emission typically reaches values of 10^−4^ to 10^−3^ for photon energies up to 10 keV^60^.

Given the low-Z sample material and the rather low compression in the experiments proposed in this work, here we assess the diagnostic potential using such “thermal” X-ray sources. In the proposed setup, radiographic imaging would be performed “on-axis”, with the imaging axis co-aligned with the cylinder axis of the LAPLAS target. This allows probing through the compressed low-Z sample without obstruction from the surrounding high-Z pusher. A schematic of the proposed setup is shown in Fig. 13 (top). Given the isotropic emission of the laser-driven plasma, the backlighter foil will need to be located rather close to the LAPLAS target (here 20 mm), to ensure a sufficient number of X-ray photons per resolution element at the target. We note that at this close proximity to the ion focus, the fluence is comparable to that heating the pusher, which would prematurely destroy the backlighter foil. Thus, this backlighting scheme is only feasible with annular beams with a negligible ion-fluence on-axis.

We have generated synthetic radiographs by performing raytracing calculations through the density distributions predicted by the hydrodynamic calculations reported in this paper. The ray sampling takes into account the expected source size typical for nanosecond laser-produced plasmas (here we have used a FWHM of 100 μm). We have assumed a laser pulse of 200 J energy (wavelength 527 nm) with nanosecond pulse duration, i.e. the laser parameters projected for the laser system to be operational for first plasma physics experiments at FAIR. Assuming a X-ray conversion efficiency of 10^−4^ this would yield 7 × 10^12^ photons at 9 keV photon energy (Zn He-alpha). At this photon energy, state-of-the-art direct detection X-ray pixel detectors like the Jungfrau detector^61^ reach near 100 % quantum efficiency, single-photon sensitivity and a high dynamic range. The detector will be placed safely outside the path of the ion beam by deflecting the transmitted X-rays with a suitable X-ray crystal. Using a highly-annealed pyrolytic graphite crystal (see e.g. ^62^) would result in a deflection angle of 24°, much larger than the ion beam focus angle of approx. 100 mrad. At a distance of 250 mm, the diameter of the ion beam will have increased to approx. 25 mm, resulting in a more than 600 times lower fluence than at the sample. Given the maximum predicted heating of the carbon sample by the on-axis fluence of 8000 K, we expect negligible heating of the X-ray crystal. It has been shown that graphite retains its high reflectivity to temperatures well above 1000 K^63^. With the detector placed at a distance of 0.5 m (resulting in a magnification of 0.5 m/20 mm = 25), a 100 μm pixel would subtend a solid angle of 3 × 10^−9^ and thus collect on average approximately 22,000 photons. Attenuation of the X-rays inside the sample is calculated using tabulated opacities^64^. The uncompressed 4 mm long sample has an areal density of 0.88 g/cm^2^, resulting in a transmission of 6.9 % for 9 keV photons. Upon compression, the areal density on-axis reaches up to 1.54 g/cm^2^, causing a significant drop in transmission to about 0.9 %. The number of detected photons in each pixels is then determined including the shot noise from the finite number of photons per pixel and the statistics of the photon absorption in the sample in order to obtain a realistic prediction of the expected image quality.

Figure 13a and b show the simulated radiographic images of the initial target and after compression at a time of 270 ns, respectively. The images clearly show the high-Z pusher wall moving in. While the edge is significantly blurred due to the extended X-ray source, the good contrast in the images would still allow to determine the wall position, thereby measuring the final sample diameter, while several measurements at different times would provide the implosion velocity. The transmission through the sample center is directly related to the on-axis areal density. Given the negligible axial rarefaction at either end of the sample, this provides a direct determination of the density. Figure 13c shows lineouts (width of one pixel) through the radiographic images. Near the center we find a pixel-to-pixel signal-to-noise ratio of 14 for the compressed sample (down from 40 for the initial target). This can be further improved when averaging over a number of adjacent pixel assuming a homogeneous density distribution. These results demonstrate that valuable information can be obtained from radiographic imaging, even when using long-pulse laser driven thermal backlighter sources.

## Conclusions

In this paper we report numerical simulations of implosion of a carbon sample employing a special LAPLAS scheme that is driven by an intense uranium beam having a circular focal spot. This scheme simplifies the problem as it does not require use of a wobbler to generate a hollow beam with an annular focal spot. The 2D hydrodynamic code, BIG2, is used to carry out the simulations. A wide range of beam parameters that correspond to the design parameters of the SIS100 beam at the FAIR facility, are used. These include a bunch intensity of 5 × 10^10^, 10^11^, 2 × 10^11^ and 3 × 10^11^ uranium ions, respectively. The bunch length is 200 ns (foot-to-foot), with a parabolic temporal intensity profile. Different values of the focal spot size characterized with FWHM of the Gaussian intensity distribution, including 2, 2.5, 3, 3.5 and 4 mm, respectively, are considered.

The target is a multi-layered cylinder comprised of a carbon sample that is enclosed in a tungsten shell. Different values of the initial sample radius, R$_{si}$ = 200, 300, 400 and 500 $\mu$m, respectively, are used, whereas the outer cylinder radius is considered to be 5 mm. Various target lengths that include, 3, 4, 5, 6, 7 mm, respectively, are assumed. These studies indicate that over the considered wide beam and target parameter range, it is possible to generate the extreme physical conditions in the carbon sample that exist deep below the surface of the carbon rich extrasolar planets , where carbon is expected to exist in the diamond phase. These experiments will therefore be very useful to understand the structure and evolution of these planets. Moreover, it will also be possible to study the formation conditions of the special type of diamonds with stacking disorders named diaphites, discovered at different asteroid impact sites. The techniques that will be used to diagnose these experiments, are also briefly discussed.

## Data Availability

The data that support the finding of this study are available within the article.
